# Meta-research: Evaluation and Improvement of Research Methods and Practices

**DOI:** 10.1371/journal.pbio.1002264

**Published:** 2015-10-02

**Authors:** John P. A. Ioannidis, Daniele Fanelli, Debbie Drake Dunne, Steven N. Goodman

**Affiliations:** Meta-Research Innovation Center at Stanford (METRICS), Stanford University, Stanford, California, United States of America

## Abstract

As the scientific enterprise has grown in size and diversity, we need empirical evidence on the research process to test and apply interventions that make it more efficient and its results more reliable. Meta-research is an evolving scientific discipline that aims to evaluate and improve research practices. It includes thematic areas of methods, reporting, reproducibility, evaluation, and incentives (how to do, report, verify, correct, and reward science). Much work is already done in this growing field, but efforts to-date are fragmented. We provide a map of ongoing efforts and discuss plans for connecting the multiple meta-research efforts across science worldwide.

## Abstract 2

Meta-research is the science of evaluating how to do, report, verify, correct, and reward scientific investigation. Many initiatives focus on these important issues, and more can be done.

## Why Perform Research on Research?

Throughout the history of science, leading scientists have endeavoured to theorize and conduct research on fundamental aspects of the scientific method and to identify ways to implement it most efficiently. While focused subject matter questions and discoveries attract attention and accolades, the machinery of science relies greatly on progressive refinement of methods and improvement of theory verification processes. The large majority of the most used articles across science are about methodology [1], and many scientific prizes are awarded for the development of techniques (e.g., Nobel prizes for PCR and MRI). Studying the scientific method in itself empirically is thus a topic of great potential value. Even though the scientific method has solid theoretical foundations and a long track record of successes, it is a continuing challenge to know how its basic principles (“systematic observation, measurement, and experiment, and the formulation, testing, and modification of hypotheses”, according to the Oxford English Dictionary) should be applied optimally in ways that can lead to faster, better, more accurate, and ultimately more useful results. In biomedical research in particular, lives can depend on the efficiency with which reliable evidence is generated and used.

This challenge is increasing, in parallel with the clear success of the scientific enterprise, which has grown in both size and diversity. Several million new research papers are published annually, and the number of publishing authors in 1996–2011 exceeded, according to one estimate, 15 million [2]. Across biomedicine, the number of articles published is increasing, and the acceleration is becoming more prominent over time, e.g., Pubmed has indexed (as of July 6, 2015) 435,302 items published in 1994, 636,951 items published in 2004 (1.46-times those published 10 years ago in 1994), and 1,182,143 items published in 2014 (1.85-times those published 10 years ago in 2004). Moreover, the wide availability of big data and the accumulation of huge amounts of data (available often online) create new opportunities and bias threats for the production of scientific knowledge, and they may challenge existing notions of data sharing, data ownership, research planning, collaboration, and replication. Mounting evidence suggests that the reproducibility of research findings in biomedicine and other disciplines is alarmingly low, that the scientific process is frustratingly inefficient, and that the number of false-positives in the literature exceedingly high; this may be a by-product of the growing complexity and multiplicity of observations, hypotheses, tests, and modifications thereof [3–8]. In biomedicine, it has been estimated that 85% of the invested effort and resources are wasted because of a diverse array of inefficiencies [3].

The geometric growth of the scientific corpus allows new opportunities for studying research practices with large-scale evidence and for testing empirically their effectiveness at producing the most reliable evidence. While one can theorize about biases (e.g., publication bias, reporting bias, selection bias, confounding, etc.), it is now possible to examine them across multiple studies and to think about ways to prevent or correct them. Many ideas and solutions have been proposed about how to strengthen the research record, including, but not limited to, registration of studies, improved standards for reporting of research, wider (even public) availability of raw data and protocols, sharing, prespecification of hypotheses, improved statistical tools and choice of rules of inference, reproducibility checks and adoption of a replication culture, team work and consortia-building, minimization of conflicts of interest, and more [9].

## A Hot but Fragmented Scientific Discipline

Many scientists are already working on these solutions, because they realize that improving methods and practices within research is integral to their quest for better and more reliable research results in their own field. Some fields could benefit from the knowledge and experience that has accumulated in other fields where various solutions have been tested and applied. However, many scientists do not closely track what is happening in fields different from their own, even within their own broad discipline. Thus, independent fragmented efforts are made to solve what are intrinsically similar challenges, albeit in different manifestations and in different environments. It is possible that the best solutions may not be the same for all fields, e.g., preregistration of experimental protocols may not serve the ends of exploratory “blue sky” science in the same way it does for clinical trials. However, one needs to see the big picture to identify the relevant similarities and differences. A research effort is needed that cuts across all disciplines, drawing from a wide range of methodologies and theoretical frameworks, and yet shares a common objective; that of helping science progress faster by conducting scientific research on research itself. This is the field of meta-research.

## What Is Included in the Discipline of Meta-research?

As for all disciplines, multiple classifications are possible, and categories are inevitably overlapping. We believe it convenient to categorize meta-research into five major areas of interest: Methods, Reporting, Reproducibility, Evaluation, and Incentives. These correspond, respectively, with how to perform, communicate, verify, evaluate, and reward research. Table 1 lists the issues that are covered under each theme and some delineation of specific interests. Many scientists are currently working on these various aspects of meta-research, motivated by the common objective to improve the scientific enterprise, but tend to do it in methodologic or disciplinary silos; unlike a physical and organic chemist, who both recognize they are chemists, these reformers within science may not recognize that they are all working within the domain of meta-research.

**Table 1:** Major themes covered by meta-research.

Meta-research area	Specific interests (nonexhaustive list)
**Methods**: "performing research"—study design, methods, statistics, research synthesis, collaboration, and ethics	Biases and questionable practices in conducting research, methods to reduce such biases, meta-analysis, research synthesis, integration of evidence, crossdesign synthesis, collaborative team science and consortia, research integrity and ethics
**Reporting**: "communicating research"—reporting standards, study registration, disclosing conflicts of interest, information to patients, public, and policy-makers	Biases and questionable practices in reporting, explaining, disseminating and popularizing research, conflicts of interest disclosure and management, study registration and other bias-prevention measures, and methods to monitor and reduce such issues
**Reproducibility**: "verifying research"—sharing data and methods, repeatability, replicability, reproducibility, and self-correction	Obstacles to sharing data and methods, replication studies, replicability and reproducibility of published research, methods to improve them, effectiveness of correction and self-correction of the literature, and methods to improve them
**Evaluation**: "evaluating research"—prepublication peer review, postpublication peer review, research funding criteria, and other means of evaluating scientific quality	Effectiveness, costs, and benefits of old and new approaches to peer review and other science assessment methods, and methods to improve them
**Incentives**: "rewarding research": promotion criteria, rewards, and penalties in research evaluation for individuals, teams, and institutions	Accuracy, effectiveness, costs, and benefits of old and new approaches to ranking and evaluating the performance, quality, value of research, individuals, teams, and institutions

Given the types of questions addressed, meta-research interfaces with many other established disciplines. These include, but are not limited to, history and philosophy of science (epistemology), psychology and sociology of science, statistics, data science, informatics, evidence-based medicine (and evidence-based “X” in general), research synthesis methods (e.g., meta-analysis), journalology, scientometrics and bibliometrics, organizational and operations research, ethics, research integrity and accountability research, communication sciences, policy research, and behavioural economics.

Meta-research includes both theoretical and empirical investigation. The former uses analytical as well as computational methods, the latter yields descriptive evidence (e.g., surveys of biases in a given field), association and correlation observational analyses, and intervention studies (e.g., randomized trials assessing whether one research practice leads to better outcomes than another). Meta-research involves taking a bird’s eye view of science. For example, single meta-analyses that synthesize evidence on multiple studies on a specific question of interest are not within the primary remit of meta-research. However, the combination of data from multiple meta-analyses on multiple topics (“meta-epidemiology”) may offer insights about how common and how consistent certain biases are across a large field or multiple fields. This emphasis on the broader picture is typical of many meta-research investigations.

**Fig 1:**
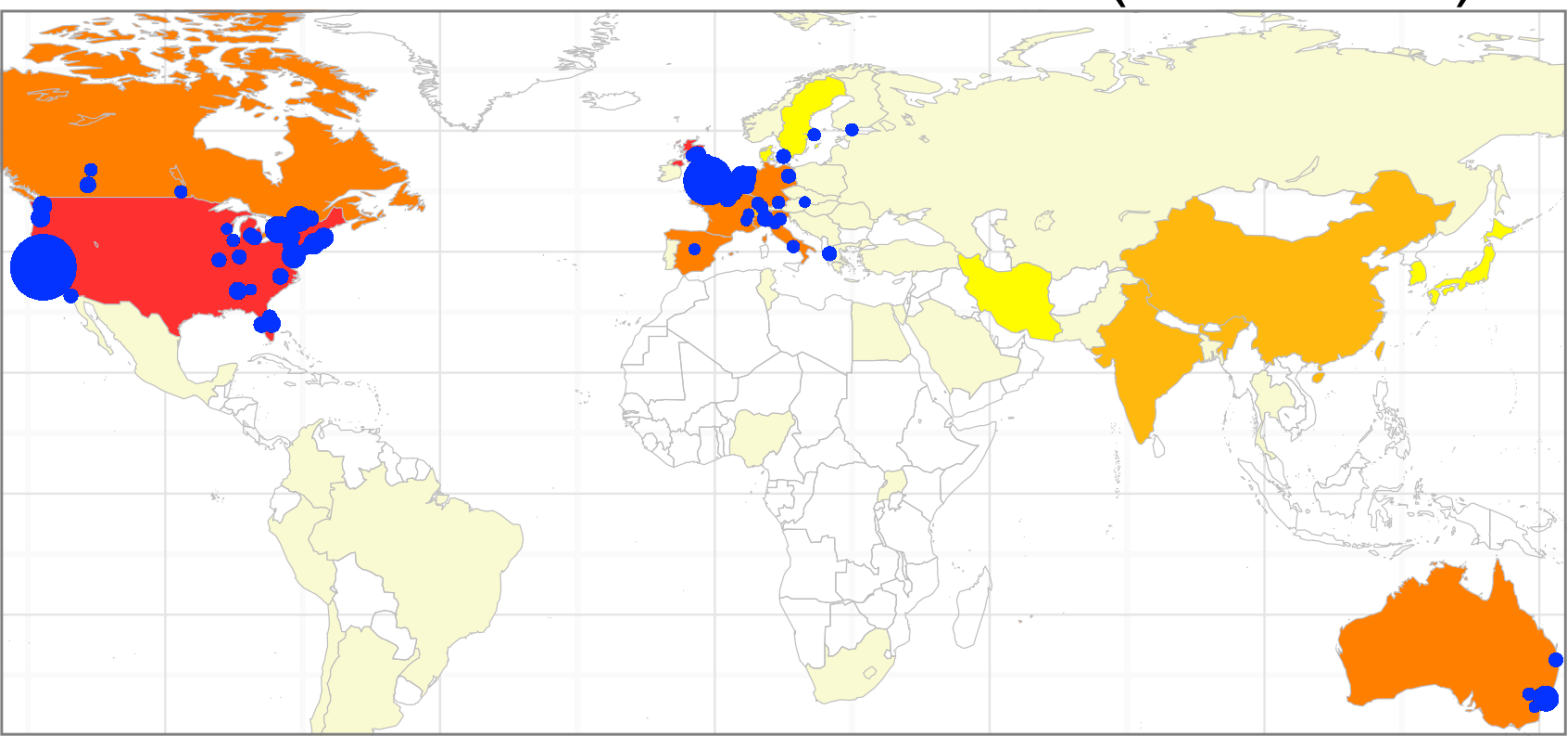
Number of meta-research–related publications registered by the Scopus database between January 1 and May 16 2015, by country of corresponding author and by affiliation of any coauthor. Countries are attributed based on corresponding or first author address (legend, from light yellow to red, respectively, to 1–5, 5–10, 10–20, 20–50, 50–230 publications). Blue dots indicate the 100 institutions most frequently listed amongst coauthors’ addresses. Dot size is proportional to number of papers (range: 2–37). Papers were selected for inclusion from an initial list of 1,422 papers retrieved from the Scopus database using a combination of search terms aimed at capturing the core areas described in Table 1. Of the 851 records selected for inclusion, country or affiliation data could not be retrieved for 102 Scopus records, which therefore are not included in the map. Search terms, literature lists, and further details are available at metrics.stanford.edu. The map and plots therein were generated anew, using the packages ggmap and ggplot2 implemented in the open source statistical software R. *Image Credit: Daniele Fanelli*

We are in the process of mapping the influential meta-research literature and identifying the key players in this burgeoning field. By an iterative process of search and manual inspection, we have compiled a search string comprising 79 terms (keywords, sentences, author identifiers) that capture with good efficiency the five thematic areas described above. A search in the Scopus database using these terms, followed by manual inspection and cross-checked selection by two of the authors (JPAI and DF), identified 851 meta-research–relevant publications (out of a starting list of 1,422) that have been published, across all disciplines, in the period January 1–May 16 2015 alone. Around three quarters of these records (*n* = 610) are classified by Scopus as research articles, conference papers, or reviews, and the rest as editorial material or letters. This preliminary “photograph” of the field suggests that meta-research is a growing and truly global enterprise (Fig 1), even though our sample is likely to underestimate the true extent of the field, since it is not fully sensitive yet to detect all relevant papers, given the very wide variety of disciplines and nomenclature involved. Identifying the boundaries of any discipline, let alone those of a highly cross-disciplinary field, is a dynamic and somewhat arbitrary process, which requires continuous updates and refinements. Therefore, a list of meta-research literature and details of the search strategy used will be posted on metrics.stanford.edu, where they will be regularly updated, expanded, and refined over time.

## Meta-research–Related Initiatives Worldwide

Table 2 shows an illustrative list of some existing initiatives that aim to address different portions of the meta-research agenda. This list is not complete, and the number of initiatives may continue to grow fast. The table aims only to give the reader a sense of the breadth of the various efforts that are ongoing. Many initiatives were launched only within the last few years. This diversity suggests that an effort is needed to better define and connect this rapidly growing discipline.

**Table 2:** A nonexhaustive list of initiatives that address various meta-research themes^*^.

Initiative	Area of work (website)
METHODS	
Cochrane Collaboration	Systematic reviews of health care (cochrane.org)
Campbell Collaboration	Systematic reviews of social science (campbellcollaboration.org)
James Lind Library	Evolution of fair tests of treatment (jameslindlibrary.org)
Society for Clinical Trials	Clinical trials (sctweb.org)
SRSM	Methods for research synthesis (srsm.org)
BioSharing	Standards for biology, natural, and life sciences (biosharing.org)
Human Proteome Project	Collaboration center for proteome (thehpp.org)
NCPRE	Research ethics (ethicscenter.csl.illinois.edu)
REPORTING	
ClinicalTrials.gov	Clinical trials registration (clinicaltrials.gov)
EQUATOR network	Reporting standards for research (equator-network.org)
Sense About Science	Communicating research in public (senseaboutscience.org)
Health News Reviews	Expert review of science news stories (healthnewsreview.org)
REPRODUCIBILITY	
Center for Open Science	Open science in psychology and more (centerforopenscience.org)
BITSS	Transparency in social sciences (bitss.org)
BPS	Best practices in social sciences (bps.stanford.edu)
Political Science Replication	Reproducibility in political science (politicalsciencereplication.com)
YODA	Sharing data from clinical research (yoda.yale.edu)
Neurovault	Data repository for PET and MRI maps (neurovault.org)
OpenfMRI	fMRI data repository (openfmri.org)
NIH repositories, examples:	
dbGAP	Raw data on genotype and phenotype (ncbi.nlm.nih.gov/gap)
GEO	Functional genomics repository (ncbi.nlm.nih.gov/geo)
Science Exchange	Reproducibility checks (validation.scienceexchange.com)
EVALUATION	
Peer Review Congress	Evidence on peer review (peerreviewcongress.org)
Center for Scientific Integrity	Tracking retractions of scientific articles (retractionwatch.com/the-center-for-scientific-integrity
PubMed Commons	Postpublication comments (ncbi.nlm.nih.gov/pubmedcommons)
ArXiv	Preprint article repository (arxiv.org)
ICMJE	Standards for journal publishing (icmje.org)
COPE	Journal publication ethics (publicationethics.org)
PubPeer	Peer comments on research (pubpeer.com)
PEERE	New models for peer review (www.peere.org)
INCENTIVES	
REWARD	Reducing waste and rewarding diligence in research (researchwaste.net)
AAAS	Science policy (aaas.org)
ICSU	International science policy (icsu.org)

*for clarity, each initiative has been grouped under one of the five themes of Table 1, but several of these initiatives cater to more than one of the five themes

AAAS: American Association for the Advancement of Science; BITSS: Berkeley Initiative for Transparency in the Social Sciences; BPS: Best Practices in Science; COPE: Committee on Publication Ethics; dbGAP: Database on Genotypes and Phenotypes; EQUATOR: Enhancing the quality and transparency of reporting; GEO: Gene Expression Omnibus; ICMJE: International Committee of Medical Journal Editors; ICSU: International Council for Science; NCPRE: National Center for Professional and Research Ethics; NIH: National Institutes of Health; REWARD: Reduce research waste and reward diligence; SRSM: Society for Research Synthesis Methodology; YODA: Yale University Open Data Access.

The Meta-Research Innovation Center at Stanford (METRICS) is one such effort that we have undertaken, with the primary objective to connect the disparate elements of this field and enhance their synergy and collective efficiency towards the goal of improving published research. It does this through primary research and creation of a research and policy-focused network of meta-researchers around the world. METRICS has recruited a large number of faculty, from multiple disciplines within and outside biomedicine, and scholars and graduate students at Stanford, has created a seed grant research program to support innovative research ideas in this area, and has started building further this meta-research community through speaker series, curriculum development, regular workshops, and other events.

A major challenge for this center is to connect the much larger global community of meta-researchers and related stakeholders. As part of building and supporting this network, we plan to create an interactive online platform to inform and connect researchers working on these themes. No single center can cover this vast field alone, so we see METRICS as a partner and facilitator of the multiple other related scientific endeavors. A biannual meeting will help bring together scientists working in distant fields who are interested in improving research practices. The first of these meetings will take place at Stanford on November 19–20, 2015.

A central goal of this community is to provide evidence-based guidance on policy initiatives to improve research quality. Such evidence should come not only from observational but also from experimental studies and through dialogue and engagement with key stakeholders from the public and private sectors. The most ambitious and durable transformations will likely require considerable realignment of the reward and incentive system in science. Funding agencies, institutional leaders, scientific journals, and the mass media will all be important partners in ensuring that the best science is designed, conducted, analyzed, published, disseminated, and ultimately rewarded.

## Better Education in Better Research Practices

A strong educational curriculum and the development of training materials to equip researchers with the knowledge of best scientific practices will also be a critical component in accomplishing these goals. There is a need to train meta-researchers, in the same way we train immunologists or biologists or computer scientists, and not just expect that some scientists will keep finding their way into meta-research in somewhat random fashion. There is also a need to educate practicing scientists, not just meta-research specialists, on the importance of methods and rigorous, reproducible research practices. Most disciplinary training focuses on learning topical subject matter facts and technical skills that are field-specific and that can have a short half-life. Conversely, there is little training of future investigators and little continuing education of mature investigators on fundamental principles of research methods and practices. Beyond scientists, other key stakeholders, including media, journal editors, and funders can be educated on best research practices.

Building such an educational curriculum may require integrating best research practices modules with required Responsible Conduct of Research training and evaluations and collaborating with other scholars to share best practices and facilitate shared learning, and creating online courses in specific methods areas. NIH recently issued a Request for Proposals for online training in this domain. Even the general public would benefit from exposure to these issues, and many activated consumer networks (e.g., Project LEAD, sponsored by the National Breast Cancer Coalition [http://www.breastcancerdeadline2020.org/get-involved/training/project-lead/], Consumers United for Evidence-based Healthcare [http://us.cochrane.org/CUE], and PCORI’s Patient Powered Research Networks [http://www.pcornet.org/patient-powered-research-networks/]) are leading the way in patient and consumer scientific engagement and education.

## Who Will Fund Research on Research?

Funding all these efforts requires a substantial investment. Until recently, the few large-scale initiatives in this space, such as the Cochrane Collaboration, were based mostly on volunteering of idealistic individuals who cared about science and high-quality evidence. Most of that effort was invested on performing systematic reviews on topical questions of interest (e.g., learning about whether a specific drug works and by how much), although this led inevitably to concerns about larger meta-research issues like bias, methods, and reproducibility across studies. Most funding agencies have organized themselves into sections based on topical focus rather than widely applicable, cross disciplinary methods. This disease or discipline-specific paradigm does not lend itself to solving problems that cut across science more generally. Until now, mostly a few private foundations have been championing the cause of meta-research to improve research quality. However, it is encouraging to see several public funders (e.g., NIH [10] and PCORI [www.pcori.org/blog/open-science-pcoris-efforts-make-study-results-and-data-more-widely-available] among others) recognizing the need to support such efforts and to eventually generate and apply scientific evidence on scientific investigation, including how they themselves should function.

## Abbreviation

METRICS: Meta-Research Innovation Center at Stanford
